# Gross motor skills and social behavior in childhood: a public health perspective on their developmental association

**DOI:** 10.3389/fpsyg.2025.1714785

**Published:** 2025-11-25

**Authors:** Jiaying Zhang, Yulan Zhou, Renke He, Xiandan Ye, Xi Chen

**Affiliations:** College of Physical Education and Health Sciences, Zhejiang Normal University, Jinhua, China

**Keywords:** social behavior, locomotor skills, object control skills, developmental stages, public health

## Abstract

**Background:**

Gross motor skills are hypothesized to contribute to social behavior in childhood, yet it remains unclear whether specific motor skill types are uniquely associated with social behavior at different ages. The present study examines the distinct and developmentally-specific relationships among locomotor skills, object control skills, and social behavior during three stages: early (3–5 years), middle (6–8 years), and late childhood (9–10 years).

**Methods:**

This investigation employed a cross-sectional design with a sample of 578 Chinese children (51.9% male, 48.1% female) recruited from kindergarten and primary school settings. The Test of Gross Motor Development–Third Edition (TGMD-3) was used to obtain an objective measure of gross motor skills. To evaluate social behavior, the teacher-completed version of the Social Skills Improvement System Rating Scales (SSIS) was administered. The relationships between variables were examined through hierarchical regression analyses, which controlled for the potential confounding effects of sex, body mass index (BMI), and physical activity.

**Results:**

Compared to the 3–5 year group, children aged 6–8 and 9–10 years displayed significantly greater proficiency in locomotor skills and received higher ratings on measures of social behavior. A steady, progressive improvement in object control skills was also evident with each older age group. According to regression analyses, object control skills served as a significant positive predictor for social behavior at every stage of development (*β* = 0.224–0.419, *p* < 0.01). Conversely, locomotor skills showed a significant relationship with social behavior solely in the early childhood period (*β* = 0.453, *p* < 0.001), with no such links found during middle or late childhood.

**Conclusion:**

Object control skills appear to be a consistent correlate of social behavior throughout childhood, whereas locomotor skills are particularly relevant in the early years. These results highlight the promise of implementing targeted interventions, such as customized physical education curricula and organized play sessions, that focus on specific motor competences as a public health initiative to promote social skill growth. To optimize efficacy, the architecture of such programs must be developmentally appropriate and responsive to age-related variations.

## Introduction

Social behavior involves the capacity to perceive, interpret, and respond to the actions, intentions, and dispositions of other people (Cacioppo and Berntson, 2005). The proficient acquisition of core competencies—including emotional regulation and peer cooperation—establishes a vital foundation for healthy peer interactions and achievement in academic settings (Denham and Brown, 2010). In contrast, children who demonstrate maladaptive social behaviors are at significant risk for negative outcomes. Such behavioral challenges, encompassing rule defiance, social isolation, irritability, and aggression, are associated with poor social adjustment, conduct disorders, dysfunctional interpersonal relationships, and a greater likelihood of delinquent behavior (Ladd and Kochenderfer-Ladd, 2002). Recent analyses of childhood social behavior have identified several alarming trends. Firstly, the incidence of social-behavioral problems has undergone a pronounced increase. Research demonstrates that a substantial proportion of young children now exhibit significant social difficulties, impairments in regulating emotions, and aggressive conduct (Collishaw and Stephan, 2015). Secondly, the presentation of these problems has become more complex and diverse. This complexity is reflected in the common diagnosis of clinical disorders like Attention-Deficit/Hyperactivity Disorder and Oppositional Defiant Disorder in both preschool and school-aged children (American Psychiatric Association, 2013). Consequently, from a public health standpoint, the identification of malleable factors that support positive social development is a critical objective for preventative measures and health promotion campaigns.

Among these potential factors, gross motor skills, the observable manifestation of broader motor competence, represent a promising yet underexplored target for population-level intervention. Grounded in developmental theory that posits the integrated growth of physical, cognitive, and social domains (Payne and Isaacs, 2016), motor competence facilitates a child’s interaction with their environment, thereby generating opportunities for social learning and psychological growth (Kenny et al., 2016). Empirical evidence confirms that gross motor skills is positively associated with social outcomes across diverse populations, including children with autism spectrum disorder (ASD) (Holloway et al., 2018; Wang et al., 2022) and developmental coordination disorder (Kim et al., 2016). Critically, longitudinal studies involving typically developing children demonstrate that early gross motor skills predict later social functioning, including reduced behavioral problems and lower risk of peer victimization (Bart et al., 2007; Øksendal et al., 2022), underscoring their potential role in shaping developmental trajectories.

Gross motor skills are typically divided into two core domains: locomotor skills (e.g., running and jumping), pertaining to the movement of the body through space, and object control skills (e.g., throwing and catching), which concern the handling of items. A growing area of investigation proposes that these distinct motor domains may correlate with social functioning through different pathways. Object control skills provide a critical foundation for cooperative games and sports. Competence in these skills facilitates successful entry into peer activities, fosters teamwork, and helps build social status (MacDonald et al., 2016). Supporting this mechanistic link, studies of school-aged children with ASD have found that better object control skills are correlated with more developed social skills (MacDonald et al., 2013). This correlation is also observed in typically developing children; in one study, preschoolers’ object control skills predicted more positive social interactions in the classroom setting (MacDonald et al., 2016), while another study connected it to improve on-task behavior among school-aged children from lower-income backgrounds (Burns et al., 2019). Crucially, intervention studies offer causal support; a ball skills program for 7–11-year-olds resulted in concurrent improvements in both object control and social behaviors (Westendorp et al., 2014). Furthermore, locomotor skills are fundamental for enabling children to physically access and navigate their social environment, for instance, by allowing them to approach peer groups or participate in chase games, thereby reducing the risk of social isolation (Smyth and Anderson, 2011). Supporting their broad social significance, a recent preschool intervention demonstrated that improvements in locomotor abilities were directly correlated with enhanced social conduct (Brian et al., 2024).

A primary limitation in existing research, which is also a crucial consideration for public health strategies, involves the potential for the association between gross motor skills and social conduct to fluctuate throughout the developmental period. Social environments and expectations mature in parallel with motoric capabilities. To illustrate, autonomous movement fundamentally alters the ways in which toddlers participate in social interactions (Thelen, 2000). Conversely, games with established rules, which are common later in childhood, depend on more advanced motor competencies for effective inclusion among peers (Staples and Reid, 2010). Although these expectations are rooted in theory, empirical investigations that directly examine developmental stage as a moderating variable in the motor-social link remain scarce.

Informed by Piaget’s theory of cognitive development, we posit that motor and social domains are fundamentally connected via a child’s active exploration of their environment. Furthermore, we propose that the type of gross motor skill most critical for social success evolves throughout development, reflecting the changing motor demands of the peer play landscape (Piaget, 1952). In early childhood, locomotor skills (e.g., running and jumping) are paramount, as they are the primary tools for engaging in the chase-based, free-running play that dominates this period (Goodway et al., 2010). As children enter middle childhood, the social world expands to include more structured activities; both locomotor and object control skills (e.g., throwing and catching) become integral for social standing and effective involvement in complex games. Deficits in either domain during this phase can heighten the risk of peer rejection or the emergence of aggressive conduct (Staples and Reid, 2010). By late childhood, the peer culture becomes heavily organized around rule-based games and sports, shifting the primary emphasis to object control skills as the central requirement for full participation and acceptance. While locomotor skills remain necessary, they often assume a secondary, supportive role within these activities (Harter, 2012).

While we acknowledge the potential for bidirectional relationships and the influence of confounding factors like cognitive function and socioeconomic status, the current study focuses specifically on how two fundamental gross motor skill categories—locomotor and object control—predict social behavior across childhood. A central aim is to investigate how these associations are moderated by developmental stage, thereby exploring the evolving nature of motor-social linkages throughout this critical period. Drawing on Piaget’s model, we advance a differentiated hypothesis: we predict that locomotor skills will demonstrate a stronger association with social behavior during early childhood (ages 3–5), whereas object control skills are expected to be more predictive in late childhood (ages 9–10). For the middle childhood period (ages 6–8), we hypothesize that both locomotor and object control skills will be of approximately equal importance for the development of social competencies. By pinpointing the distinct developmental mechanisms by which gross motor skills facilitate social behavior, this research seeks to generate evidence crucial for shaping the scheduling, focus, and implementation of population-level public health strategies designed to enhance psychosocial outcomes.

## Methods

### Participants

This research was carried out during the spring academic semester (February to June 2025) within public kindergarten and primary school settings located in Jinhua City, Zhejiang Province. The sampling strategy employed a convenience approach, selecting one kindergarten and one primary school, both affiliated with a local university. As these institutions predominantly enroll children of university employees, the participant pool was characterized by high sociodemographic homogeneity, particularly in terms of parental educational attainment and household socioeconomic status.

From the chosen schools, two classes were randomly selected from each of the lower, middle, and upper kindergarten levels, and three classes were chosen at random from each grade level (1st to 4th) in the primary school. This sampling procedure yielded a preliminary participant group of 717 children, aged 3 to 10, drawn from a total of 18 classes. Before the commencement of data collection, comprehensive informed consent forms, which outlined the study’s purpose and protocols, were provided to all parents or legal guardians. Signed consent was successfully secured for every child involved.

All 717 participants completed assessments of their gross motor skills, and their respective head teachers provided evaluations of social behavior for each child. The subsequent data cleaning process led to the exclusion of 78 children due to incomplete motor assessment data, 50 due to invalid accelerometer readings, and 11 due to absent questionnaire returns. Consequently, the final analytic cohort comprised 578 children (51.9% male, 48.1% female). A comparative attrition analysis was conducted between the final analytical sample (*n* = 578) and participants excluded for incomplete data (*n* = 139). This analysis, which compared key demographic variables (age, sex), revealed no statistically significant differences (*p* > 0.05), indicating that the data were likely missing at random. The sample was distributed across age groups as follows: 179 children (31.0%) were in the 3–5 year range, 196 children (33.9%) were 6–8 years old, and 203 children (35.1%) were in the 9–10 year age group.

### Measures

#### Social behavior

Children’s social behavior was evaluated using the teacher form of the Social Skills Improvement System Rating Scales (SSIS-RS). This well-validated instrument is designed to measure core social behaviors that contribute to social competence in individuals aged 3–18 and is recognized for its strong psychometric properties (Gresham and Elliott, 2008). The assessment tool comprises seven distinct domains: Assertion (7 items), Communication (7 items), Cooperation (6 items), Empathy (6 items), Engagement (7 items), Responsibility (6 items), and Self-Control (7 items). For each item, teachers indicated the frequency of the observed behavior on a 4-point Likert-type scale, ranging from 1 (“never”) to 4 (“almost always”). A composite raw score was derived from the sum of all items, yielding a potential range of 0 to 184, where elevated scores reflect more adaptive and positive social functioning. The present analysis employed these total raw scores.

#### Gross motor skills

The assessment of gross motor skills was conducted using the Test of Gross Motor Development–Third Edition (TGMD-3) (Ulrich, 2019). This is a widely adopted, process-oriented assessment that analyzes the qualitative aspects of movement patterns and has established excellent reliability and validity for use with children between 3 and 10 years of age (Ma et al., 2022; Webster and Ulrich, 2017). The TGMD-3 evaluates six distinct locomotor skills (running, galloping, hopping, skipping, horizontal jumping, and sliding) and seven object-control skills (two-hand striking, one-hand striking of a self-bounced ball, dribbling, two-hand catching, kicking, overhand throwing, and underhand tossing). All evaluations adhered to standardized TGMD-3 procedures: each skill was first explained verbally and then demonstrated by a certified assessor, after which the child completed two practice trials. A secondary demonstration was provided if a child exhibited a misunderstanding or executed the skill incorrectly. All performances were recorded on video for subsequent scoring by trained evaluators based on standardized checklists. Each skill is assessed against three to five specific performance criteria per trial. A score of 1 was allocated for each criterion successfully demonstrated and 0 for those not met. The raw score for each skill was calculated by summing the scores from its two trials. The total subtest scores for Locomotor (range: 0–46) and Object Control (range: 0–54) were derived by summing the raw scores of all constituent skills within each subtest.

#### Physical activity

Objectively measured physical activity, encompassing light physical activity (LPA) and moderate-to-vigorous physical activity (MVPA), was captured using ActiGraph wGT3X-BT accelerometers. This device is a well-established and reliable tool for quantifying activity levels in child populations (Robusto and Trost, 2012). Trained research assistants provided instructions on correct placement and affixed the monitor to the right anterior hip line using an adjustable elastic belt. Children were instructed to wear the device for five full consecutive days during all waking hours, removing it only for water-based activities or sleep. To mitigate potential reactivity, data from the first day were excluded from analysis. The subsequent 4 days of data, which included a minimum of two weekdays and two weekend days, were retained for processing. Data analysis was conducted using ActiLife software (v.6.5), employing a 15-s epoch interval and a sampling frequency of 30 Hz. A valid day of recording was defined as a minimum of 10 h of wear time within the period of 6:00 a.m. to 10:00 p.m. Participants were required to have a minimum of three valid days, including at least one weekend day, for inclusion. Periods of non-wear were automatically identified as spans of 20 consecutive minutes or more with zero activity counts (Choi et al., 2011). To classify activity intensity, population-specific cut-points validated for Chinese children were applied: LPA was defined as 100–2,799 counts per minute (CPM), and MVPA was defined as ≥2,800 CPM (Zhu et al., 2013). The final variables of interest were the average daily minutes spent in LPA and MVPA, which were utilized as covariates in statistical models.

#### Body mass index

Anthropometric measurements were taken with participants wearing lightweight indoor clothing and no footwear. Standing height was determined using a calibrated portable stadiometer, with values recorded to the nearest 0.1 centimeter. Body weight was measured with a precision digital scale, recorded to the nearest 0.1 kilogram. For each child, body mass index (BMI) was subsequently computed according to the conventional formula: weight in kilograms divided by the square of height in meters (kg/m^2^).

### Data collection

This study’s protocol was approved by the University’s Institutional Review Board and the administrative bodies of the participating schools. The first author, along with eight research assistants specializing in sports pedagogy, conducted the data collection over a two-week period at each participating school. A sequential approach was employed: anthropometric data (height and weight), demographic details (age, sex), and gross motor skills (via TGMD-3) were gathered in the first week. The subsequent week was dedicated to collecting accelerometer-based physical activity data and teacher-completed social behavior assessments (SSIS-RS).

Gross motor skill evaluations were administered in sessions with small groups of three to four children. Four research assistants, who had undergone standardized TGMD-3 training led by a certified expert, conducted all assessments and recorded them on video. These video files were subsequently scored in duplicate by trained evaluators. To determine inter-rater reliability, a randomly chosen subset of 20% of the videos was scored independently by two raters. The resulting intraclass correlation coefficients were excellent, reaching 0.91 for the locomotor subscale and 0.95 for the object-control subscale, thereby exceeding established benchmarks for agreement (Barnett et al., 2014).

For the physical activity monitoring phase, each participant was issued a uniquely identified accelerometer and instructed to wear it during their typical daily schedule. Research assistants provided initial demonstrations on correct placement and performed periodic checks during school hours to verify adherence to wearing protocols. Parents were given both written guidelines and verbal explanations to help them re-secure the monitor each morning. Height and weight were measured for all children who yielded valid accelerometer data. Sex was self-reported by the children, while age information was confirmed through parental report.

The head teacher of each involved classroom completed the SSIS-RS for every student in their class. To evaluate the temporal stability of these measures, the same rating scale was re-administered to teachers following a two-week hiatus. The internal consistency of the subscales, as measured by Cronbach’s alpha, ranged from 0.80 to 0.95, denoting excellent reliability.

### Data analysis

All analyses were conducted using IBM SPSS Statistics Version 26.0, with the significance threshold set at *p* < 0.05. Prior to inferential testing, the normality of all variables was assessed by examining skewness and kurtosis; as no significant deviations from normality were detected, parametric methods were employed. Descriptive statistics—including means, standard deviations, and frequencies—were computed to summarize participant characteristics, gross motor skills, social behavior, and physical activity levels. Developmental stage differences across key variables were examined using a series of one-way analyses of variance (ANOVA). For any significant main effects, *post hoc* comparisons were conducted using the Least Significant Difference (LSD) test. Effect sizes were estimated using partial eta-squared (*η*^2^), interpreted as small (0.01), medium (0.06), or large (0.14) according to conventional guidelines (Cohen, 1988). Bivariate relationships among variables were assessed using Pearson correlation coefficients, calculated separately for each of the three developmental stages (3–5, 6–8, and 9–10 years). To evaluate the unique contribution of gross motor skills to social behavior, a two-step hierarchical regression analysis was performed separately for each developmental stage. In the first step, sex, BMI, LPA, and MVPA were entered as covariates based on existing literature (Barnett et al., 2016; Burton et al., 2023). In the second step, locomotor and object control skills were added to determine whether they explained significant additional variance in social behavior scores beyond the covariates.

## Results

### Description of variables by developmental stage

Descriptive statistics for participant demographics, gross motor skills, social behavior, and physical activity levels (LPA, MVPA) across the three developmental stages are summarized in Table 1. The sample’s sex distribution was relatively even within each age cohort: 3–5 years (53.1% male, 46.9% female), 6–8 years (51.5% male, 48.5% female), and 9–10 years (51.2% male, 48.8% female). Analysis revealed a significant main effect of age group on BMI (*F*[2, 575] = 22.96, *p* < 0.001, *η^2^* = 0.072). According to *post hoc* LSD tests, BMI was notably lower in the 3–5 and 6–8 year cohorts compared to the 9–10 year group. Age-related disparities were also evident in physical activity. A significant effect was found for LPA (*F*[2, 575] = 56.43, *p* < 0.001, *η^2^* = 0.341), with children in the two younger groups participating in significantly more LPA than their older peers. A similar, significant downward trend emerged for MVPA across successive age groups (*F*[2, 575] = 18.90, *p* < 0.001, *η^2^* = 0.257). Gross motor skills exhibited considerable age-related progression. Significant main effects were identified for both locomotor (*F*[2, 575] = 15.63, *p* < 0.001, *η^2^* = 0.047) and object control skills (*F*[2, 575] = 68.51, *p* < 0.001, *η^2^* = 0.449), indicating improvement with age. Follow-up tests specified that locomotor skills were more developed in the 6–8 and 9–10 year groups than in the 3–5 year group. Object control skills, however, showed significant gains with each successive developmental stage. Finally, social behavior scores also differed significantly across the age groups (*F*[2, 575] = 25.12, *p* < 0.001, *η^2^* = 0.074). *Post hoc* comparisons using the LSD test indicated that the youngest children (3–5 years) had significantly lower social behavior scores than those in the two older groups, between which no significant difference was found.

**Table 1:** Descriptive characteristics of the study sample.

Variables	3–5 years (*n* = 179)	6–8 years (*n* = 196)	9–10 years (*n* = 203)	*F*	*η^2^*
Sex					
Male	95 (53.1%)	101 (51.5%)	104 (51.2%)	−	−
Female	84 (46.9%)	95 (48.5%)	99 (48.8%)	−	−
Age	4.53 (0.5)	7.11 (0.8)	9.36 (0.52)	116.39	0.903
BMI	15.48 (1.79)	15.71 (2.09)	17.08 (3.09)	22.96	0.072
PA					
LPA (min)	310.29 (55.08)	303.46 (57.59)	262.81 (59.27)	56.43	0.341
MVPA (min)	60.40 (26.94)	54.71 (20.51)	45.00 (12.99)	18.90	0.257
Gross motor skill					
Locomotor skills	31.81 (8.08)	34.95 (7.15)	35.11 (2.72)	15.63	0.047
Object-control skills	22.51 (5.79)	33.38 (7.66)	38.78 (4.13)	68.51	0.449
Social behavior	107.22 (18.36)	116.18 (14.93)	118.04 (15.04)	25.12	0.074

LPA, light physical activity; MVPA, moderate to vigorous physical activity.

### Bivariate correlations

Bivariate correlations for all variables, calculated separately for each age group, are presented in Table 2. Significant associations were found between sex and locomotor skills in 3–5-year-olds (*r* = 0.490, *p* < 0.05) and 9–10-year-olds (*r* = 0.465, *p* < 0.01), and between sex and object control skills in 3–5-year-olds (*r* = 0.594, *p* < 0.01) and 6–8-year-olds (*r* = 0.456, *p* < 0.01). BMI was significantly correlated with locomotor skills, object control skills, and social behavior, but only among the 6–8 and 9–10-year-old children (*r* = 0.413–0.689). LPA and MVPA showed significant correlations with social behavior across all developmental stages (*r* = 0.363–0.874). In addition, LPA were correlated with both locomotor (3–5-year-olds: *r* = 0.339, *p* < 0.01; 6–8-year-olds: *r* = 0.425, *p* < 0.01) and object control skills (3–5-year-olds: *r* = 0.436, *p* < 0.01; 6–8-year-olds: *r* = 0.547, *p* < 0.01) in 3–5 and 6–8-year-olds and locomotor skills in 9–10-year-olds (*r* = 0.381, *p* < 0.05). Similarly, MVPA was associated with both motor skill types in 3–5-year-olds (locomotor: *r* = 0.588, *p* < 0.01; object control: *r* = 0.555, *p* < 0.01), 6–8-year-olds (locomotor: *r* = 0.530, *p* < 0.01; object control: *r* = 0.554, *p* < 0.05), and 9–10-year-olds (locomotor: *r* = 0.227, *p* < 0.05; object control: *r* = 0.343, *p* < 0.05). Crucially, a strong and consistent relationship was observed between gross motor skills and social behavior. Object control skills were significantly correlated with social behavior across all age groups (*r* = 0.684–0.760, *p* < 0.01). Locomotor skills were also significantly correlated with social behavior in 3–5-year-olds (*r* = 0.534, *p* < 0.01) and 6–8-year-olds (*r* = 0.508, *p* < 0.05), with one exception: locomotor skills were not significantly associated with social behavior in the 9–10-year-old group (*r* = 0.084, *p* > 0.05).

**Table 2:** Correlation matrix for outcome and control variables for different age groups.

Variables	1	2	3	4	5	6	7
3–5 years							
1. Sex^a^	−						
2. BMI	0.065	−					
3. LPA	0.206	0.159	−				
4. MVPA	0.326	0.077	0.641^**^	−			
5. Locomotor skills	0.490^*^	0.089	0.339^**^	0.588^**^	−		
6. Object-control skills	0.594^**^	0.237	0.436^**^	0.555^**^	0.619^**^	−	
7. Social behavior	0.068	0.167	0.682^**^	0.775^**^	0.534^**^	0.684^**^	−
6–8 years							
1. Sex^a^	−						
2. BMI	0.506^**^	−					
3. LPA	0.330^*^	0.649^*^	−				
4. MVPA	0.783^**^	0.100	0.648^**^	−			
5. Locomotor skills	0.042	0.446^*^	0.425^**^	0.530^**^	−		
6. Object-control skills	0.456^**^	0.602^**^	0.547^**^	0.554^*^	0.681^**^	−	
7. Social behavior	0.030	0.549^*^	0.363^*^	0.874^**^	0.508^*^	0.760^**^	−
9–10 years							
1. Sex^a^	−						
2. BMI	0.446^*^	−					
3. LPA	0.697^**^	0.589^**^	−				
4. MVPA	0.586^**^	0.542^*^	0.614^**^	−			
5. Locomotor skills	0.465^**^	0.502^**^	0.381^*^	0.227^*^	−		
6. Object-control skills	0.039	0.413^*^	0.077	0.343^*^	0.102	−	
7. Social behavior	0.035	0.689^**^	0.729^**^	0.793^**^	0.084	0.654^**^	−

^a^0 = female, 1 = male.

LPA, light physical activity; MVPA, moderate to vigorous physical activity.

*p < 0.05, **p < 0.01.

### Hierarchical regression analysis

Hierarchical regression analyses were conducted to assess the unique contribution of gross motor skills (locomotor and object control) to social behavior across developmental stages, after controlling for sex, BMI, LPA, and MVPA. As presented in Table 3, among the 3–5-year-old age group, the control variables (Model 1) explained 13.3% of the variance in social behavior (*Adj. R^2^* = 0.114), with both LPA (*β* = 0.450, *p* < 0.001) and MVPA (*β* = 0.357, *p* < 0.001) as significant predictors. The addition of gross motor skills in Model 2 significantly increased the explained variance by 20.2% (*∆R^2^*), resulting in a total adjusted variance explained of 31.6%. In this final model, both locomotor (*β* = 0.453, *p* < 0.001) and object control skills (*β* = 0.238, *p* < 0.001) were significant positive predictors of social behavior.

**Table 3:** Hierarchical regression analyses predicting social behavior from control variables and gross motor skills.

Developmental stage	Variables	*B*	*SE*	*β*	*P*	*R^2^_adj_*	*∆R^2^*
3–5 years							
Model 1						0.114	0.133
	Sex^a^	1.111	0.563	0.028	0.893		
	BMI	1.256	1.946	0.060	0.259		
	LPA	2.895	0.539	0.450	<0.001		
	MVPA	3.104	0.884	0.357	<0.001		
Model 2						0.316	0.202
	Sex^a^	1.124	0.652	0.105	0.596		
	BMI	1.721	1.796	0.080	0.059		
	LPA	2.534	0.695	0.215	<0.001		
	MVPA	2.449	0.697	0.315	<0.001		
	Locomotor skills	1.768	0.467	0.453	<0.001		
	Object-control skills	1.970	0.529	0.238	<0.001		
6–8 years							
Model 1						0.112	0.243
	Sex^a^	1.762	1.738	0.061	0.312		
	BMI	0.593	0.377	0.113	0.117		
	LPA	0.609	0.232	0.209	0.003		
	MVPA	0.836	0.283	0.336	0.002		
Model 2						0.459	0.347
	Sex^a^	1.522	1.712	0.052	0.142		
	BMI	0.313	0.377	0.063	0.407		
	LPA	0.676	0.222	0.276	0.010		
	MVPA	0.753	0.278	0.353	0.002		
	Locomotor skills	0.233	0.434	0.233	0.592		
	Object-control skills	1.019	0.120	0.419	<0.001		
9–10 years							
Model 1						0.216	0.350
	Sex^a^	2.410	1.353	0.080	0.307		
	BMI	1.167	0.372	0.167	0.005		
	LPA	1.028	0.318	0.228	0.003		
	MVPA	0.764	0.256	0.464	<0.001		
Model 2						0.369	0.153
	Sex^a^	1.956	1.430	0.056	0.422		
	BMI	0.817	0.337	0.171	0.020		
	LPA	0.759	0.280	0.259	0.007		
	MVPA	1.264	0.356	0.584	0.001		
	Locomotor skills	0.021	0.140	0.021	0.882		
	Object-control skills	0.924	0.317	0.224	0.008		

The adjusted *R*^2^ and change in *R*^2^ (∆*R*^2^) are provided for each model as well as the unstandardized beta coefficients (B), standard errors (SE), standardized beta (β) and statistical significance values for all predictor variables, including the control covariates.

LPA, light physical activity; MVPA, moderate to vigorous physical activity.

^a^0 = female, 1 = male.

For the 6–8-year-old cohort, Model 1 accounted for 24.3% of the variance (*Adj. R^2^* = 0.112), with LPA (*β* = 0.209, *p* = 0.003) and MVPA (*β* = 0.336, *p* = 0.002) as significant predictors. Model 2, which included gross motor skills, explained a total of 45.9% of the variance, representing a significant increase of 34.7%. Object control skills emerged as a strong significant predictor (*β* = 0.419, *p* < 0.001), alongside the persistent effects of LPA (*β* = 0.276, *p* = 0.010) and MVPA (*β* = 0.353, *p* = 0.002).

In the 9–10-year-old group, the control variables in Model 1 explained 35.0% of the variance (*Adj. R^2^* = 0.216), with significant contributions from BMI (*β* = 0.167, *p* = 0.005), LPA (*β* = 0.228, *p* = 0.003), and MVPA (*β* = 0.464, *p* < 0.001). The inclusion of gross motor skills in Model 2 accounted for a significant additional 15.3% of the variance, for a total adjusted variance explained of 36.9%. In the final model, object control skills remained a significant predictor (*β* = 0.224, *p* = 0.008), along with BMI (*β* = 0.171, *p* = 0.020), LPA (*β* = 0.259, *p* = 0.007), and MVPA (*β* = 0.584, *p* = 0.001).

## Discussion

This study investigated the developmental associations between distinct gross motor skill domains and social behavior, testing a differentiated hypothesis that their relative importance would shift across childhood. The results provide partial support for this hypothesis, revealing a nuanced pattern: while object control skills demonstrated a robust and consistent association with social behavior across all age groups, the significant contribution of locomotor skills was developmentally specific, limited to the early childhood period (3–5 years).

### Gross motor skills and social behavior across childhood

Descriptive analyses revealed expected developmental trajectories in both motor and social domains. Locomotor skills were significantly less developed among 3–5-year-olds compared to older children, while object control skills improved progressively with age. This pattern aligns with global developmental trends in gross motor skills (Bolger et al., 2021). The significant improvement in locomotor skills with age reflects known neurophysiological maturation, including neural myelination and the development of strength and balance. The high variability in these skills among 3–5-year-olds allows them to act as a key differentiator for social engagement (Webb et al., 2001). In contrast, object control skills, which depend on more complex inter-segmental coordination and specific practice, demonstrate a steadier, cumulative developmental trajectory. Consequently, they maintain their potential as a social differentiator across a broader age span (Logan et al., 2012).

Similarly, Children aged 3–5 years demonstrated significantly lower social behavior scores compared to those aged 6–8 and 9–10 years. This pattern reflects a normative developmental trajectory in social competence, marking the transition from a primarily egocentric perspective to that of a socially aware agent capable of understanding norms, engaging in cooperation, and demonstrating empathy (Eisenberg et al., 2006). This evolution is supported by advances in cognitive and emotional capacities that are still emergent in early childhood. Children aged 3–5 are only beginning to develop theory of mind—the understanding that others may hold beliefs, desires, and intentions different from their own. Concurrently, their ability to regulate emotions remains limited. Consequently, strong feelings such as frustration or disappointment, which exceed their regulatory capacity, are often expressed through externalizing behaviors like tantrums or aggression, which can disrupt social interactions and elicit negative responses from peers (Denham, 2006). Furthermore, the social environment of 3–5-year-olds differs substantially from that of older children. Interactions are often facilitated by adults, occur primarily through parallel play, and feature fluid, loosely structured peer relationships (Eggum-Wilkens et al., 2014). As children grow older, they enter more complex and cooperative social settings—such as structured games and peer groups—that provide increased opportunities to practice and refine social skills, thereby contributing to higher observed scores in social behavior.

### Locomotor skills and social behavior

The study revealed a developmentally specific association between locomotor skills and social behavior, which was significant only in the 3–5-year-old group. This finding suggests that the contribution of locomotor proficiency to social behavior is particularly salient during early childhood. The period from 3 to 5 years of age represents a sensitive period for acquiring gross motor skills like running, jumping, and climbing (Gallahue et al., 2012). Proficiency in these skills directly enables participation in peer play—such as chasing games or hide-and-seek—which serves as a primary medium for social interaction. Children with more advanced locomotor abilities are often better equipped to initiate and sustain these physically oriented social exchanges, thereby gaining more opportunities for social engagement (Pagani and Messier, 2012; Piek et al., 2008). This direct relationship, however, attenuates in middle childhood for two key reasons. Our analyses indicated that locomotor skills were significantly more developed and less variable among 6–8 and 9–10-year-olds compared to the younger group, suggesting that as these skills become mastered by most children, their power to differentiate social outcomes diminishes. Furthermore, social interaction patterns undergo a significant shift during this period. With advances in linguistic and cognitive abilities, socialization becomes less dependent on physical play and increasingly mediated through verbal communication, rule-based games (e.g., board games), and organized activities (Harter, 2012; Staples and Reid, 2010). In these contexts, social strategies grow more sophisticated; children can employ humor, conversation, or shared interests to form relationships, reducing the relative importance of motor competence (Wellman, 2014). Thus, while locomotor skills provide a crucial foundation for early social interaction, their direct influence attenuates as children’s social and cognitive worlds expand.

### Object control skills and social behavior

In contrast to the developmentally specific role of locomotor skills, object control skills demonstrated a robust and enduring association with social behavior. Regression analyses revealed that object control skills were a significant and positive predictor of social behavior across all three age groups (3–5, 6–8, and 9–10 years). This consistent association aligns with previous research underscoring the robust link between object manipulation and social development in childhood (MacDonald et al., 2013; Pagani and Messier, 2012; Westendorp et al., 2014). This persistent relationship may be attributed to shared neurocognitive mechanisms, particularly those involving action understanding and social-cognitive processing. Object control actions, such as throwing or passing, are believed to engage neural systems, potentially including the mirror neuron system, which are involved in action understanding and intention perception (Rizzolatti and Craighero, 2004). It is hypothesized that this motor-based understanding of others’ actions may provide a foundational substrate for the development of more abstract social-cognitive abilities. Furthermore, the complex cognitive processes required for object manipulation—including spatial perception, motor planning (praxis), and feedback-based online correction—rely on a network involving the cerebellum and parietal cortex (Grafton, 2009). Notably, these executive and visuomotor integration functions share neural substrates (e.g., the prefrontal cortex, posterior parietal cortex, and cerebellum) with, and may thereby support, the development of social-cognitive abilities like theory of mind, which is mediated by the prefrontal cortex (Leslie, 1987). From a social learning perspective, proficiency in object control enhances a child’s sense of agency and self-efficacy within their physical and social environments (Frey, 2008). This confidence is critical for social engagement. Collaborative object-oriented activities, such as building with blocks or playing team sports, provide natural contexts for children to practice and internalize prosocial behaviors—including sharing, turn-taking, and cooperative problem-solving—thereby directly facilitating the development of social skills.

Although object control skills significantly predicted social behavior across all age groups, they accounted for the greatest proportion of variance among 6–8-year-old children. The peak explanatory power of object control skills at age 6–8 emerges from a perfect confluence of developmental readiness and social-environmental demand. According to Piaget’s theory, children in this concrete operational stage begin to comprehend and adhere to rules in structured games. Within this context, proficient object control skills—such as throwing or passing accurately—are not merely motor actions but essential for executing game strategies and demonstrating cooperative intelligence (Bjorklund and Pellegrini, 2000). A successful pass, for example, requires both motor competence and social-cognitive abilities like anticipating a teammate’s movement and reading the opposition, thereby providing a direct medium for behaviors like cooperation and communication to be expressed and reinforced. Concurrently, the social ecology of 6–8-year-olds undergoes a significant shift from family-centered to peer-oriented interactions, where gaining acceptance and forming friendships become paramount (Piaget, 1965). Advanced object control skills can directly elevate social status, create leadership opportunities, and expand social networks (Burton et al., 2023), making motor proficiency a particularly powerful contributor to social success during this period. In contrast, for 3–5-year-olds, social interactions are still largely scaffolded by adults and revolve around less structured, parallel play, muting the exclusive social advantage of object control. By ages 9–10, peer groups become more stabilized and interests diversify. Children may increasingly value domains like academics, arts, or digital games alongside physical play (Brown and Larson, 2009; Rubin et al., 2015). While object control skills remain relevant, their unique explanatory power diminishes as social success becomes influenced by a wider array of factors, including shared interests, humor, and academic competence.

### Limitations and future research

Several limitations of this study should be considered when interpreting its findings. First, the participant sample was recruited from a single geographic region and exhibited relatively homogeneous socioeconomic characteristics, which may limit the generalizability of the results. Future research should seek to include more diverse populations across varied cultural and socioeconomic contexts to enhance the external validity and broaden the applicability of the findings. Second, the assessment of social behavior relied predominantly on teacher reports, which are susceptible to subjective bias and may not fully capture the complexity of children’s social interactions in different settings. Future studies would benefit from incorporating multi-method and multi-informant approaches—such as direct behavioral observations in naturalistic (e.g., playground) or semi-structured environments, parent reports, and peer nominations—to provide a more comprehensive and objective measure of social behavior. Third, the cross-sectional nature of this study precludes causal inferences regarding the relationships between gross motor skills and social behavior. To elucidate the directional and mechanistic pathways underlying these associations, future research should employ longitudinal designs that track developmental trajectories over time. Additionally, intervention studies designed to enhance motor skills could provide experimental evidence to determine whether improvements in motor competence lead to positive changes in social outcomes, thereby strengthening claims of causality.

### Implications and recommendations

From a population health perspective, these findings affirm that gross motor skills represent a malleable target for improving psychosocial health, yet they also demonstrate that interventions must be both developmentally timed and domain-specific. For early childhood (ages 3–5), educational and recreational curricula should prioritize locomotor skill development through activities like obstacle courses, tag games, and dancing. This focus directly supports the fundamental, physically-oriented social interactions characteristic of this developmental period. For middle and late childhood (beginning around age 6), structured opportunities to master object control skills become crucial. Physical education and recess should be deliberately designed to progressively build competence in throwing, catching, and kicking. This is best achieved through cooperative, inclusive tasks that emphasize participation and skill-acquisition over outright competition. Finally, screening for motor competence—particularly for persistent object control difficulties—should be integrated into holistic support plans for children experiencing social isolation or behavioral challenges. Such screening can identify a key, and often overlooked, modifiable factor contributing to a child’s psychosocial struggles.

## Data Availability

The raw data supporting the conclusions of this article will be made available by the authors, without undue reservation.

## References

[ref1] American Psychiatric Association (2013). Diagnostic and statistical manual of mental disorders. 5th Edn. Arlington, VA: American Psychiatric Publishing.

[ref2] Barnett L. M. Lai S. K. Veldman S. L. C. Hardy L. L. Cliff D. P. Morgan P. J. . (2016). Correlates of gross motor competence in children and adolescents: a systematic review and meta-analysis. Sports Med. 46, 1663–1688. doi: 10.1007/s40279-016-0495-z, PMID: 26894274 PMC5055571

[ref3] Barnett L. M. Minto C. Lander N. Hardy L. L. (2014). Interrater reliability assessment using the test of gross motor Development-2. J. Sci. Med. Sport 17, 667–670. doi: 10.1016/j.jsams.2013.09.013, PMID: 24211133

[ref4] Bart O. Hajami D. Bar-Haim Y. (2007). Predicting school adjustment from motor abilities in kindergarten. Infant Child Dev. 16, 597–615. doi: 10.1002/icd.514

[ref5] Bjorklund D. F. Pellegrini A. D. (2000). Child development and evolutionary psychology. Child Dev. 71, 1687–1708. doi: 10.1111/1467-8624.00258, PMID: 11194266

[ref6] Bolger L. E. Bolger L. A. O’Neill C. Coughlan E. O’Brien W. Lacey S. . (2021). Global levels of fundamental motor skills in children: a systematic review. J. Sports Sci. 39, 717–753. doi: 10.1080/02640414.2020.1841405, PMID: 33377417

[ref7] Brian A. Munn E. E. Abrams T. C. Case L. Taunton M. S. Stribing A. . (2024). Skipping with PAX: evaluating the effects of a dual-component intervention on gross motor skill and social–emotional development. J. Mot. Learn. Dev. 12, 228–246. doi: 10.1123/jmld.2024-0023

[ref8] Brown B. B. Larson J. (2009). “Peer relationships in adolescence” in Handbook of adolescent psychology: Contextual influences on adolescent development. eds. Lerner R. M. Steinberg L., vol. 2. 3rd ed (Hoboken, NJ: Wiley).

[ref9] Burns R. D. Byun W. Brusseau T. A. (2019). Gross motor skills predict classroom behavior in lower-income children. Front Sports Act Living. 1:29. doi: 10.3389/fspor.2019.00029, PMID: 33344953 PMC7739582

[ref10] Burton A. M. Cowburn I. Thompson F. Eisenmann J. C. Nicholson B. Till K. (2023). Associations between motor competence and physical activity, physical fitness and psychosocial characteristics in adolescents: a systematic review and meta-analysis. Sports Med. 53, 2191–2256. doi: 10.1007/s40279-023-01886-1, PMID: 37542607 PMC10587315

[ref11] Cacioppo J. T. Berntson G. G. (2005). “Social neuroscience” in Social neuroscience: key readings. eds. Cacioppo J. T. Berntson G. G. (New York: Psychology Press).

[ref12] Choi L. Liu Z. Matthews C. E. Buchowski M. S. (2011). Validation of accelerometer wear and non-wear time classification algorithm. Med. Sci. Sports Exerc. 43, 357–364. doi: 10.1249/MSS.0b013e3181ed61a3, PMID: 20581716 PMC3184184

[ref13] Cohen J. (1988). Statistical power analysis for the behavioral sciences. 2nd Edn. Hillsdale, NJ: Lawrence Erlbaum Associates.

[ref14] Collishaw S. Stephan M. (2015). Annual research review: secular trends in child and adolescent mental health. J. Child Psychol. Psychiatry 56, 370–393. doi: 10.1111/jcpp.12372, PMID: 25496340

[ref15] Denham S. A. (2006). Social-emotional competence as support for school readiness: what is it and how do we assess it? Early Educ. Dev. 17, 57–89. doi: 10.1207/s15566935eed1701_4

[ref16] Denham S. A. Brown C. (2010). ‘Plays nice with others’: social–emotional learning and academic success. Early Educ. Dev. 21, 652–680. doi: 10.1080/10409289.2010.497450

[ref17] Eggum-Wilkens N. D. Fabes R. A. Castle S. Zhang L. Hanish L. D. Martin C. L. (2014). Playing with others: head start children’s peer play and relations with kindergarten school competence. Early Child Res. Q. 29, 345–356. doi: 10.1016/j.ecresq.2014.04.008, PMID: 24882941 PMC4038679

[ref18] Eisenberg N. Fabes R. A. Spinrad T. L. (2006). “Prosocial development” in Handbook of child psychology: Social, emotional, and personality development. eds. Damon W. Lerner R. M., vol. 3. 6th ed (Hoboken, NJ: John Wiley & Sons).

[ref19] Frey S. H. (2008). Tool use, communicative gesture and cerebral asymmetries in the modern human brain. Philos. Trans. R. Soc. Lond. Ser. B Biol. Sci. 363, 1951–1957. doi: 10.1098/rstb.2008.0008, PMID: 18292060 PMC2606701

[ref20] Gallahue D. L. Ozmun J. C. Goodway J. D. (2012). Understanding motor development: Infants, children, adolescents, adults. 7th Edn. New York: McGraw-Hill.

[ref21] Goodway J. D. Robinson L. E. Crowe H. (2010). Gender differences in fundamental motor skill development in disadvantaged preschoolers from two geographical regions. Res. Q. Exerc. Sport 81, 17–24. doi: 10.1080/02701367.2010.10599624, PMID: 20387395

[ref22] Grafton S. T. (2009). Embodied cognition and the simulation of action to understand others. Ann. N. Y. Acad. Sci. 1156, 97–117. doi: 10.1111/j.1749-6632.2009.04425.x, PMID: 19338505

[ref23] Gresham F. M. Elliott S. N. (2008). Social skills improvement system (SSIS) rating scales. Bloomington, MN: Pearson.

[ref24] Harter S. (2012). The construction of the self: Developmental and sociocultural foundations. 2nd Edn. New York: Guilford Press.

[ref25] Holloway J. M. Long T. M. Biasini F. (2018). Relationships between gross motor skills and social function in young boys with autism spectrum disorder. Pediatr. Phys. Ther. 30, 184–190. doi: 10.1097/PEP.0000000000000505, PMID: 29727358 PMC6013349

[ref26] Kenny L. Hill E. Hamilton A. F. (2016). The relationship between social and motor cognition in primary school age-children. Front. Psychol. 7:228. doi: 10.3389/fpsyg.2016.00228, PMID: 26941685 PMC4764733

[ref27] Kim H. Carlson A. G. Curby T. W. Winsler A. (2016). Relations among motor, social, and cognitive skills in pre-kindergarten children with developmental disabilities. Res. Dev. Disabil. 53–54, 43–60. doi: 10.1016/j.ridd.2016.01.016, PMID: 26852279

[ref28] Ladd G. W. Kochenderfer-Ladd B. (2002). Identifying victims of peer aggression from early to middle childhood: analysis of cross-informant data for concordance, estimation of relational adjustment, prevalence of victimization, and characteristics of identified victims. Psychol. Assess. 14, 74–96. doi: 10.1037/1040-3590.14.1.74, PMID: 11911051

[ref29] Leslie A. M. (1987). Pretense and representation: the origins of “theory of mind”. Psychol. Rev. 94, 412–426. doi: 10.1037/0033-295X.94.4.412

[ref30] Logan S. W. Robinson L. E. Wilson A. E. Lucas W. A. (2012). Getting the fundamentals of movement: a meta-analysis of the effectiveness of motor skill interventions in children. Child Care Health Dev. 38, 305–315. doi: 10.1111/j.1365-2214.2011.01307.x, PMID: 21880055

[ref31] Ma J. Duncan M. J. Chen S. T. Eyre E. L. J. Cai Y. (2022). Cross-cultural comparison of fundamental movement skills in 9- to 10-year-old children from England and China. Eur. Phys. Educ. Rev. 28, 519–533. doi: 10.1177/1356336X211055585

[ref32] MacDonald M. Lipscomb S. McClelland M. M. Duncan R. Becker D. Anderson K. . (2016). Relations of preschoolers’ visual-motor and object manipulation skills with executive function and social behavior. Res. Q. Exerc. Sport 87, 396–407. doi: 10.1080/02701367.2016.1229862, PMID: 27732149 PMC5549668

[ref33] MacDonald M. Lord C. Ulrich D. (2013). The relationship of motor skills and social communicative skills in school-aged children with autism spectrum disorder. Adapt. Phys. Act. Q. 30, 271–282. doi: 10.1123/apaq.30.3.271, PMID: 23860508

[ref34] Øksendal E. Brandlistuen R. E. Holte A. Wang M. V. (2022). Associations between poor gross and fine motor skills in pre-school and peer victimization concurrently and longitudinally with follow-up in school age: results from a population-based study. Br. J. Educ. Psychol. 92, 434–452. 10.1111/bjep.12464 34729762

[ref35] Pagani L. S. Messier S. (2012). Links between motor skills and indicators of school readiness at kindergarten entry in urban disadvantaged children. J. Educ. Dev. Psychol. 2, 95–107. doi: 10.5539/jedp.v2n1p95

[ref36] Payne V. G. Isaacs L. D. (2016). Human motor development: A lifespan approach. 8th Edn. New York: Routledge.

[ref37] Piaget J. (1952). The origins of intelligence in children. New York: International Universities Press.

[ref38] Piaget J. (1965). The moral judgment of the child. New York: The Free Press.

[ref39] Piek J. P. Bradbury G. S. Elsley S. C. Tate L. (2008). Motor coordination and social–emotional behaviour in preschool-aged children. Int. J. Disabil. Dev. Educ. 55, 143–151. doi: 10.1080/10349120802033592

[ref40] Rizzolatti G. Craighero L. (2004). The mirror-neuron system. Annu. Rev. Neurosci. 27, 169–192. doi: 10.1146/annurev.neuro.27.070203.144230, PMID: 15217330

[ref41] Robusto K. M. Trost S. G. (2012). Comparison of three generations of ActiGraph™ activity monitors in children and adolescents. J. Sports Sci. 30, 1429–1435. doi: 10.1080/02640414.2012.710761, PMID: 22857599 PMC3458797

[ref42] Rubin K. H. Bukowski W. M. Bowker J. C. (2015). “Children in peer groups” in Handbook of child psychology and developmental science: Ecological settings and processes. eds. Bornstein M. H. Leventhal T., vol. 4. 7th ed (Hoboken, NJ: John Wiley & Sons).

[ref43] Smyth M. M. Anderson H. I. (2011). Coping with clumsiness in the school playground: social and physical play in children with coordination impairments. Br. J. Dev. Psychol. 18, 389–413.

[ref44] Staples K. L. Reid G. (2010). Fundamental movement skills and autism spectrum disorders. J. Autism Dev. Disord. 40, 209–217. doi: 10.1007/s10803-009-0854-9, PMID: 19685284

[ref45] Thelen E. (2000). Grounded in the world: developmental origins of the embodied mind. Infancy 1, 3–28. doi: 10.1207/S15327078IN0101_02, PMID: 32680313

[ref46] Ulrich D. A. (2019). Test of gross motor development–third edition: examiner’s manual. Austin, TX: Pro-Ed.

[ref47] Wang L. A. Petrulla V. Zampella C. J. Waller R. Schultz R. T. (2022). Gross motor impairment and its relation to social skills in autism spectrum disorder: a systematic review and two meta-analyses. Psychol. Bull. 148, 273–300. doi: 10.1037/bul0000358, PMID: 35511567 PMC9894569

[ref48] Webb S. J. Monk C. S. Nelson C. A. (2001). Mechanisms of postnatal neurobiological development: implications for human development. Dev. Neuropsychol. 19, 147–171. doi: 10.1207/S15326942DN1902_2, PMID: 11530973

[ref49] Webster E. K. Ulrich D. A. (2017). Evaluation of the psychometric properties of the test of gross motor development–third edition. J. Mot. Learn. Dev. 5, 45–58. doi: 10.1123/jmld.2016-0003

[ref50] Wellman H. M. (2014). Making minds: How theory of mind develops. Oxford: Oxford University Press.

[ref51] Westendorp M. Houwen S. Hartman E. Mombarg R. Smith J. Visscher C. (2014). Effect of a ball skill intervention on children’s ball skills and cognitive functions. Med. Sci. Sports Exerc. 46, 414–422. doi: 10.1249/MSS.0b013e3182a532b3, PMID: 23872937

[ref52] Zhu Z. Chen P. Zhuang J. (2013). Intensity classification accuracy of accelerometer-measured physical activities in Chinese children and youth. Res. Q. Exerc. Sport 84, S4–S11. 24527562 10.1080/02701367.2013.850919

